# Dried Urine Spots for Quantification of C0 (Free) Carnitine Excretion: A Validated Method for Follow-Up and Confirmatory Testing of Primary Carnitine Deficiency

**DOI:** 10.3390/ijns12040084

**Published:** 2026-10-12

**Authors:** Maria van der Ham, Sacha Ferdinandusse, Mirjam Langeveld, Sabine A. Fuchs, Monique G. M. de Sain van der Velden, on behalf of the ODIN Study Group

**Affiliations:** 1 Section Metabolic Diagnostics, Department of Genetics, University Medical Center Utrecht, 3584 EA Utrecht, The Netherlands; m.vanderham-3@umcutrecht.nl; 2 Laboratory Genetic Metabolic Diseases, Department of Laboratory Medicine, Amsterdam UMC, University of Amsterdam, 1105 AZ Amsterdam, The Netherlands; s.ferdinandusse@amsterdamumc.nl; 3 Department of Endocrinology and Metabolism, Amsterdam Gastroenterology Endocrinology Metabolism (AGEM) Research Institute, Amsterdam University Medical Center, University of Amsterdam, 1105 AZ Amsterdam, The Netherlands; m.langeveld@amsterdamumc.nl; 4 Department of Metabolic Diseases, Wilhelmina Children’s Hospital, University Medical Center Utrecht, 3584 EA Utrecht, The Netherlands; s.fuchs@umcutrecht.nl

**Keywords:** dried urine spot, C0 (free) carnitine excretion, FIA-MS/MS, primary carnitine deficiency, newborn screening

## Abstract

Primary carnitine deficiency (PCD), caused by Organic Cation Transporter Novel 2 (OCTN2) deficiency, is detected by newborn screening (NBS) through low blood free carnitine (C0) concentrations. Confirmation of the diagnosis may be supported by demonstrating increased urinary C0 excretion, but urine collection in newborns is technically challenging. We developed and validated a dried urine spot (DUS) method for quantifying urinary C0 excretion as a practical, non-invasive alternative to conventional liquid urine sampling. This approach has the potential to allow parents to obtain urine at home by placing filter paper cards between the baby’s skin and a dry diaper, simplifying sample collection. C0 carnitine and creatinine were quantified in 6 mm DUS punches by flow injection analysis–tandem mass spectrometry (FIA–MS/MS) following butylation. The method was analytically validated for sensitivity, precision, stability, and agreement with liquid urine measurements. The method demonstrated excellent analytical performance, with lower limits of detection (LLOD)/lower limits of quantification (LLOQ) values of 0.02/0.07 µmol/L for C0 and 0.01/0.04 mmol/L for creatinine. Normalization of C0 to creatinine reduced variability related to sampling and punch location. Both analytes remained stable in DUS for at least 10 days at room temperature and after 11 freeze–thaw cycles. C0/creatinine obtained from DUS showed excellent agreement with liquid urine (R^2^ > 0.9967), although concentrations were consistently lower, requiring a correction factor of 1.27 to estimate concentrations equivalent to liquid urine. DUS provides a reliable and accurate alternative to liquid urine for measuring C0 excretion in newborns. This simple, home-based sampling approach facilitates non-invasive urine collection and has the potential to support follow-up and confirmatory testing of PCD.

## 1. Introduction

Primary carnitine deficiency (PCD; MIM 212140) is an autosomal recessive disorder caused by pathogenic variants in the *SLC22A5* gene encoding the Organic Cation Transporter Novel 2 (OCTN2). Defective OCTN2 impairs cellular uptake and renal tubular reabsorption of carnitine, resulting in markedly reduced blood and tissue free carnitine (C0) concentrations.

PCD is included in NBS programs in many countries, where screening is based on low C0 concentrations measured in dried blood spots (DBS). However, the positive predictive value (PPV) of NBS for PCD remains relatively low, even when referral is preceded by confirmation of persistently low C0 in a second DBS specimen [1]. Consequently, many newborns without PCD are referred to metabolic centers, increasing healthcare costs and placing an emotional burden on families [2]. Improving the specificity of follow-up testing after an abnormal NBS result is therefore an important objective.

Carnitine homeostasis in newborns depends on placental transfer, dietary intake, limited endogenous synthesis, and efficient renal tubular reabsorption. Under physiological conditions, more than 95% of filtered carnitine is reabsorbed by OCTN2 in the proximal renal tubules. In patients with PCD, defective OCTN2 function results in excessive urinary loss of C0 carnitine despite profoundly reduced plasma C0 concentrations [3,4]. Increased urinary C0 excretion, particularly in combination with low plasma C0, is therefore a characteristic biochemical feature of PCD. Recently, we demonstrated that urinary C0 excretion is a valuable biomarker for distinguishing true PCD from false-positive NBS referrals [1]. In that study, urinary C0 excretion alone reliably discriminated affected newborns from false-positive referrals and, when combined with plasma C0, substantially improved the diagnostic performance of the follow-up evaluation. These findings highlight the potential value of urinary C0 measurements as an adjunct to current NBS algorithms.

Despite its clinical utility, urine collection in newborns remains challenging, particularly outside healthcare settings. Dried urine spots (DUS) collected on filter paper provide an attractive alternative to conventional liquid urine samples. Similar to DBS, DUS facilitates home sampling, transport by regular mail, and convenient storage while maintaining analyte stability. In addition, urine collection is painless, non-invasive, and unaffected by hematocrit, making DUS particularly suitable for neonatal follow-up testing.

DUS has since been successfully used to quantify a variety of metabolites, including amino acids, α-keto acids, 3-hydroxyglutaric acid, glycosaminoglycans, bile acids, hippuric acid, creatine, pipecolic acid, *α*-aminoadipic semialdehyde, tetrasaccharide, globotriaosylceramide, and sulfatide [5,6,7,8,9,10,11,12,13,14,15,16]. However, to our knowledge, urinary C0 carnitine has not previously been quantified using DUS. Because urinary C0 concentrations are routinely normalized to creatinine to account for urine dilution, simultaneous measurement of both analytes is required.

The aim of this study was to develop and analytically validate a method for the simultaneous quantification of C0 carnitine and creatinine in DUS. With the goal of exploring the potential of DUS as a complementary matrix in an NBS screening algorithm, we placed particular emphasis on urine application, DUS processing, metabolite stability, and evaluating the agreement between results obtained from liquid urine and DUS.

## 2. Materials and Methods

### 2.1. Chemicals and Materials

L-Carnitine·HCl, acetyl chloride, and the internal standard (IS) D_3_-creatinine were purchased from Sigma-Aldrich (Zwijndrecht, The Netherlands). Creatinine and *n*-butanol were obtained from Merck (Darmstadt, Germany). The IS D_3_-carnitine (D_3_-C0) was purchased from Cambridge Isotope Laboratories (Andover, MA, USA). Methanol (MeOH) was obtained from Riedel-de Haën (Seelze, Germany), and ULC-grade acetonitrile (ACN) from Biosolve (Dieuze, France). Milli-Q water was produced in-house using a PURELAB^®^ Chorus 1 Analytical system Elga, High Wycombe, UK). DUS were prepared on Guthrie card filter papers (Whatman no. 903 Protein Saver™, formerly Schleicher & Schuell, Keene, NH, USA). Sample discs (6 mm diameter) were excised using a hand-held punch (Fiskars, Helsinki, Finland). Urine spots were visualized under ultraviolet light (Analytik Jena, Upland, CA, USA). Validation analyses were performed using specialized assays provided by ERNDIM (European Research Network for the Evaluation and Improvement of Screening, Diagnosis, and Treatment of Inherited Disorders of Metabolism).

### 2.2. Preparation of Standard Solutions

Combined calibration standards in 10% MeOH were prepared at ten concentration levels. For C0, concentrations were 0, 4, 8, 16, 32, 48, 100, 200, 400, and 800 µmol/L, while creatinine concentrations were 0, 0.5, 0.75, 1, 2, 3.5, 5, 10, 20, and 30 mmol/L.

### 2.3. Sample Collection and Processing

Human urine samples were obtained from the Section of Metabolic Diagnostics, University Medical Center Utrecht, The Netherlands. All urine samples were coded to ensure pseudoanonymity. For the preparation of DUS, liquid urine was applied to filter paper and air-dried for 4 h at ambient temperature. The DUS were stored at −20 °C in sealed plastic bags containing a desiccant until analysis.

#### 2.3.1. Control Samples

Three pooled urinary quality control (QC) samples were prepared by combining urine from five individuals per creatinine concentration level: low (<1.5 mmol/L), medium (~8 mmol/L), and high (>16 mmol/L). Aliquots of 6 µL from each pooled sample were spotted onto filter paper.

#### 2.3.2. Sample Preparation

From each DUS, a 6 mm diameter disc was punched using a hand-held punch and transferred to a pre-labeled Eppendorf tube. For liquid urine samples, 2.5 µL was used. Subsequently, 500 µL of an IS working solution (D_3_-C0, 0.3 µmol/L; D_3_-creatinine, 0.1 mmol/L) prepared in 0.004% (*v*/*v*) MilliQ in MeOH was added. DUS samples were extracted over 15 min using an ultrasonic bath. Liquid urine samples were vortex-mixed prior to analysis. An aliquot of 5 µL of the extract was dried at 40 °C and derivatized with 50 µL of butylation reagent (prepared by mixing acetyl chloride and *n*-butanol at a 1:4 (*v*/*v*) ratio, resulting in a final acetyl chloride concentration of 3 mol/L). After incubation for 15 min at 60 °C, samples were evaporated under a nitrogen stream at 40 °C and reconstituted in 250 µL of ACN. Finally, 0.1 µL of the sample was injected into the mass spectrometer.

### 2.4. FIA-MS-MS

To reduce analysis time and cost, a flow injection analysis–tandem mass spectrometry (FIA–MS/MS) method, as widely applied in NBS, was developed. FIA was performed using the bypass mode of a BSM-I-Class UPLC system coupled to an Xevo TQ Absolute mass spectrometer (Waters, Milford, CT, USA). As the mobile phase, 90% (*v*/*v*) ACN at a flow rate of 0.2 mL/min was used. The total run time per sample was 1 min, and the sample compartment was maintained at 15 °C. The sample manager used 90% (*v*/*v*) ACN as the needle wash solvent and 10% (*v*/*v*) ACN as the seal wash. Urine and DUS samples were analyzed using multiple reaction monitoring (MRM) acquisition in positive electrospray ionization (ESI) mode. The capillary voltage was set to 2 kV, the source temperature to 120 °C, and the desolvation temperature to 600 °C. Optimal spray conditions were achieved with a desolvation gas flow of 1050 L/h, while cone and nebulizer gas flows were set to 150 L/h and 300 L/h, respectively. Fragmentation was performed using a collision gas flow of 0.15 mL/min. During method development, the MRM transition for underivatized C0 (*m*/*z* 162.0 → 103.0) was temporarily added to the MS method using a cone voltage of 25 V and a collision energy of 15 eV. MRM transitions were individually optimized for each analyte by analyzing both labeled and unlabeled standards (Table 1).

### 2.5. Data Analysis/Statistical Analysis

Data acquisition, instrument control, and processing were carried out using Waters MassLynx™ software (version 4.2, SCN 977) and including TargetLynx XS (version 4.2, SCN 977). Quantification of C0 and creatinine was based on the signal intensity ratio of each analyte to its corresponding IS, with concentrations determined from the slope of the respective standard curve. Method agreement was assessed using Bland–Altman analysis, and statistical significance was evaluated with a two-tailed *t*-test, considering *p*-values < 0.05 as significant.

### 2.6. Method Development

#### 2.6.1. Butylated Versus Non-Butylated

To compare the performance of derivatized (butylated) and underivatized methods for the accurate measurement of C0 and creatinine, the highest calibration standard (800 µmol/L C0 and 30 mmol/L creatinine) was used. For the underivatized method, 2.5 µL of standard was mixed with 500 µL of IS working solution. For both the derivatized and underivatized methods, a 5 µL aliquot of the mixture was dried at 40 °C and reconstituted in 500 µL ACN.

#### 2.6.2. Visualization of Urine on Filter Paper and Spot Volume

Urine is colorless to pale, and once dried on filter paper, localization of the exact DUS position for later processing is difficult. Spot borders were initially marked with a pencil, and dried spots were visualized under UV light (254 nm), producing blue–green fluorescence. Quantitative analysis of DUS is feasible because the filter paper is designed to hold a specific amount of the sample uniformly across its surface, ensuring that a consistent quantity is extracted when punched out [17]. Urine spot areas can be converted to volumes by constructing a standard curve that relates known volumes of human urine pipetted onto filter paper. Therefore, known volumes of urine (1 µL, 2 µL, 4 µL, 6 µL, 8 µL, and 10 µL) with two different creatinine concentrations (0.6 mmol/L and 20 mmol/L) were spotted in triplicate, and the diameter was measured using a digital caliper with an accuracy of 0.2 mm (Werckmann, Trading BV, The Netherlands).

#### 2.6.3. Extraction Efficiency

To identify the optimal extraction conditions, 2.5 µL of urine was applied to filter paper, and the entire spot was cut into a 2 mL Eppendorf tube. Analytes were extracted with 0.5 mL of solvent, followed by 15 min of sonication. Solvents tested included MeOH, ACN, MeOH/ACN/Milli-Q (1:1:1 (*v*/*v*/*v*)), and MeOH/Milli-Q at varying ratios. Experiments were performed using three distinct urine samples, and analyte recovery from DUS was expressed relative to liquid urine (set at 100%). Extraction duration was further optimized using ultrasonic extraction for 5, 10, 15, 20, 30, and 45 min on DUS QC-M samples (duplicate), followed by standard processing.

#### 2.6.4. Effect of Filter Components on Analyte Ionization

To evaluate potential matrix effects from filter paper, calibration standards (2.5 µL) were spotted onto paper, dried at room temperature for 4 h, and extracted prior to analysis.

#### 2.6.5. DUS Collection and Chromatographic Effects

Proper DUS collection is critical for accurate quantitative analysis, as inadequate sampling or processing may introduce bias. Chromatographic effects (analyte redistribution on the paper) were minimized by extracting the entire spot of a known urine volume. In practice, DUS are often collected without precise volumes, leading to potential variations. To assess this, we compared two collection approaches: spotting urine onto filter paper versus dipping filter paper strips into urine (saturation method). For spotted samples, two punch locations were evaluated: center (C) and edge (E). For dipped strips, three positions along the strip (S1, S2, and S3) were tested. Control urine samples containing low, medium, and high C0 concentrations were used to ensure consistency.

### 2.7. Method Validation

Following development and optimization, the analytical procedure was fully validated, including accuracy, precision, selectivity, specificity, linearity, lower limits of quantification and detection (LLOQ and LLOD), carry-over, recovery, process efficiency, and stability. Paired DUS and liquid urine samples were analyzed to assess variation between matrices.

#### 2.7.1. Accuracy and Precision

Accuracy was determined by comparing reported concentrations in eight ERNDIM reference samples with corresponding prepared DUS and was reported as mean accuracy (%) across concentrations. Additional accuracy assessment was performed via standard addition recovery experiments. Precision was evaluated through intra- and inter-assay variation using QC-L, QC-M, and QC-H (*n* = 5 and *n* = 25, respectively), with coefficients of variation (CV%) calculated for each.

#### 2.7.2. Selectivity and Specificity

Butylated C0 was quantified using *m*/*z* 218.2 → 103.0 [C_4_H_7_O_3_]^+^ transition, with m/z 218.2 → 85 [C_4_H_5_O_2_]^+^ monitored for confirmation. Creatinine was quantified using m/z 113.9 → 44 [CH_4_N]^+^ as the primary ion and *m*/*z* 113.9 → 86 [C_3_H_8_N_3_]^+^ as the qualifier ion. The use of unique qualifier ions ensured signal specificity and minimized potential interference from co-eluting compounds.

#### 2.7.3. Linearity

Linearity for C0 and creatinine in both liquid urine and DUS was evaluated using a 14-point calibration curve (maximum concentrations of 3784 µmol/L and 126 mmol/L, respectively), with analyte-to-IS peak area ratios plotted against nominal concentrations. The origin was excluded, and no weighting was applied.

#### 2.7.4. Sensitivity and Carry-Over

LLOD and LLOQ were determined from QC-M samples across three inter-day series. LLOD and LLOQ corresponded to signal-to-noise ratios of ≥3 and ≥10, respectively. The resulting LLOD and LLOQ values were considered S/N-based sensitivity estimates and were not directly assessed as quantitative levels during method validation. Quantitative performance was evaluated within the calibration ranges described in Section 2.2. Carry-over was evaluated by injecting blank samples immediately after the highest calibrators and QC samples to detect residual signal.

#### 2.7.5. Process Efficiency

Process efficiency was assessed using pooled urine, a standard addition of ST 10 to Milli-Q (1:4), and ST 10 to urine (1:4). Aliquots of 1.9 µL and 2.5 µL were spotted, dried, excised, and processed, with corresponding liquid aliquots analyzed in parallel. Experiments were performed in triplicate, and mean counts were calculated.

#### 2.7.6. Stability

The stability of C0 and creatinine in DUS was evaluated under various storage conditions. QC-L, QC-M, and QC-H were assessed at room temperature for 1–10 days and at 40 °C for 1, 4, and 7 days. Freeze–thaw stability was tested over eleven cycles at −20 °C and ambient temperature. Processed samples were compared to day 1 to determine relative stability. Stability of processed samples was tested for 1–28 days, with samples stored at 4 °C.

#### 2.7.7. Cross-Validation

The liquid urine method was cross-validated using 41 samples against a routinely used method: C0 measured as butyl ester derivatives by the ESI-MS/MS method [18] and creatinine measured via the Jaffé colorimetric assay. Further validation of the DUS method was performed through the analysis of 102 paired DUS and liquid urine samples to evaluate matrix comparability.

## 3. Results

### 3.1. Method Development

#### 3.1.1. Butylated Versus Non-Butylated

Butylation of C0 improved peak shape and selectivity and increased sensitivity by a factor of 100, enabling accurate quantification from very small sample volumes. To allow simultaneous analysis of both target analytes in a single run, creatinine was also subjected to the derivatization procedure, despite lacking a carboxyl (-COOH) functional group.

#### 3.1.2. Spot Volume of 6 mm

A linear relationship between DUS area and applied urine volume was observed up to 10 µL (R^2^ = 0.9953). Each 6 mm diameter DUS (28.27 mm^2^) corresponded to approximately 1.9 µL of urine.

#### 3.1.3. Extraction Efficiency

**Figure 1:**
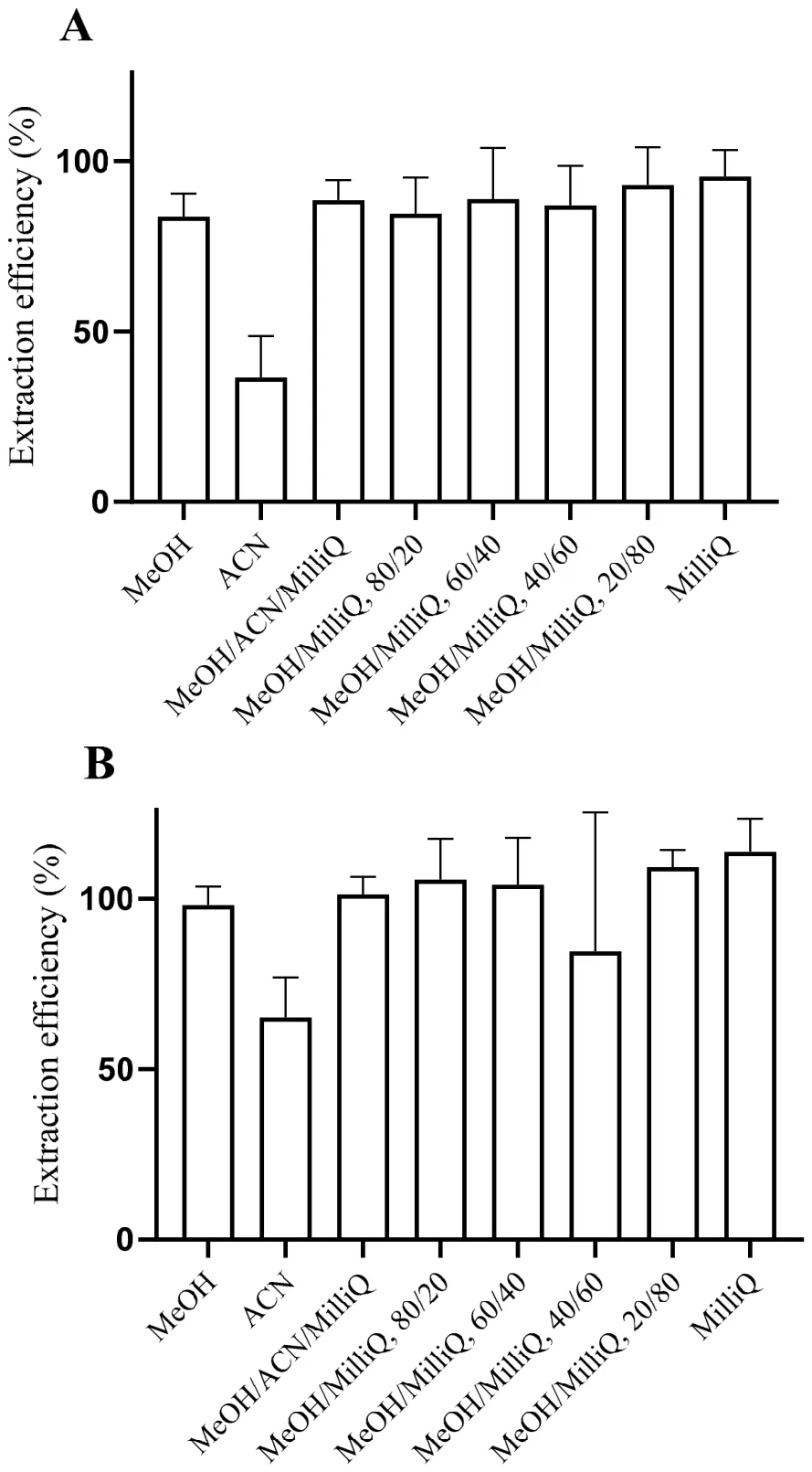
Extraction efficiency (%) using different extraction solvents. The peak area in urine was set at 100%: (**A**) C0-carnitine; (**B**) creatinine. Error bars represent the standard deviation of the three independent urine samples (*n* = 3), with each sample measured in duplicate.

Analysis of analyte responses (Figure 1) indicated that ACN provided the poorest extraction efficiency, while other tested solvents gave comparable results. Pure MeOH was chosen as an extraction solvent because of its volatility, compatibility with butylation, and its ability to efficiently extract both polar and non-polar analytes, including long-chain acylcarnitines, which are less polar and may be of interest in extended analyses of DUS. Following evaluation of extraction times, a 15-min. ultrasonic extraction yielded consistent results with minimal variability and was selected for subsequent analyses.

#### 3.1.4. Analyte Ionization

Calibration curves for both metabolites demonstrated excellent linearity, with coefficients of determination (R^2^ > 0.99). Evaluation of potential filter paper effects, performed by spotting and extracting calibration standards, showed minimal impact on analyte ionization, with slopes varying only slightly (0.09%), indicating consistent responses across the paper matrix.

#### 3.1.5. DUS Collection and Chromatographic Effects

**Figure 2:**
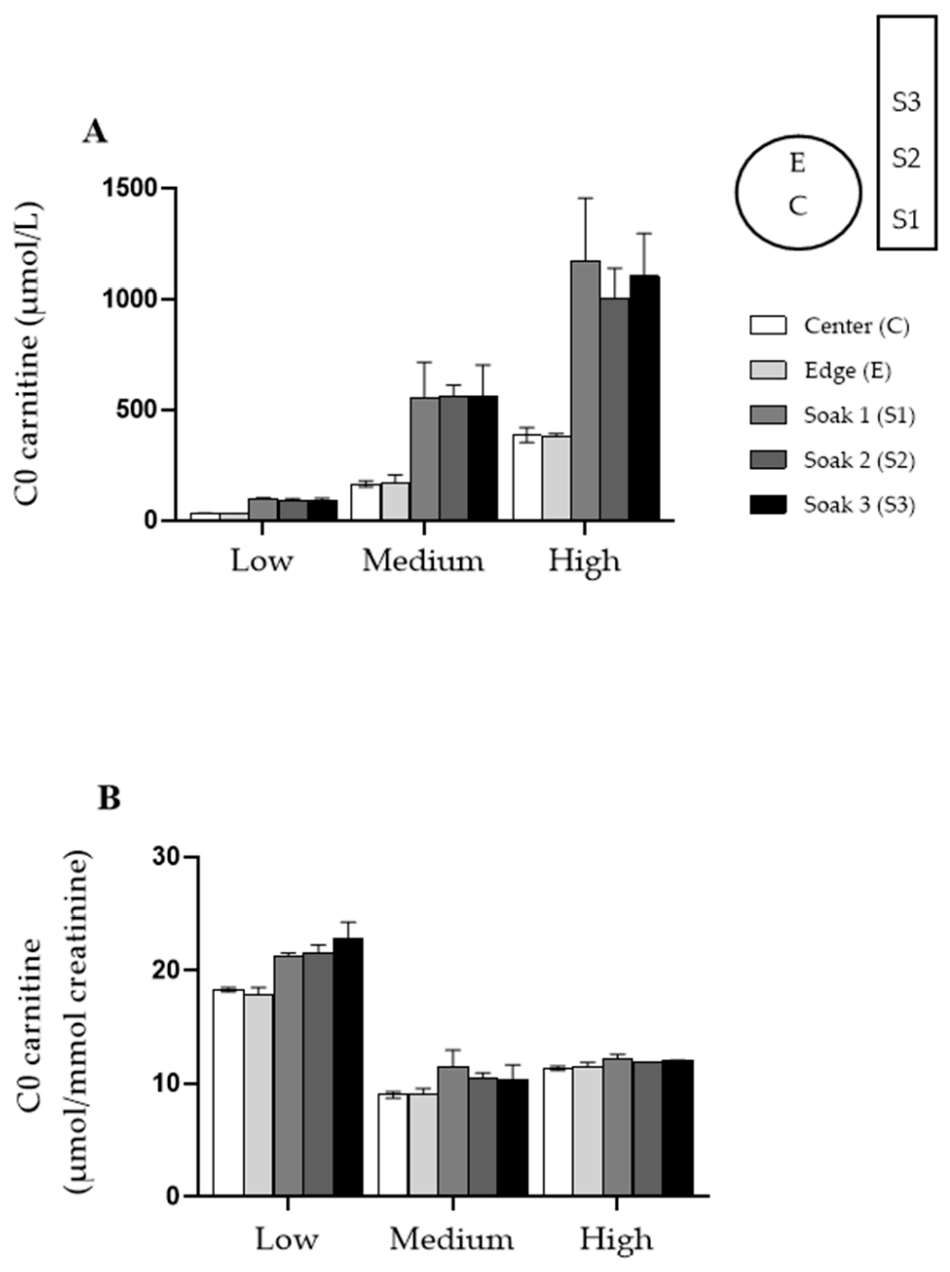
Effect of DUS collection method and punch location on C0 concentration (**A**) and C0/creatinine ratio (**B**). For spotted samples, two sub-punch locations were assessed: the center (C) and the edge (E) of the spot. For dipped strips, three punch locations (S1, S2, and S3) were evaluated along the urine-saturated strip. Measurements were obtained from three independent replicates, and error bars represent the standard deviation of *n* = 3.

For spotted samples, C0 concentrations were consistent across different punch locations. In contrast, DUS prepared by the dipping (saturation) method yielded higher C0 concentrations compared to spotted samples, consistent across all three punch locations on the dipped strips (S1, S2, and S3) (Figure 2A). Normalization to creatinine concentrations minimized these differences (Figure 2B). The resulting C0/creatinine ratios were comparable across application methods and punch locations, indicating that creatinine correction effectively compensated for volume- and dispersion-related variability.

### 3.2. Method Validation

#### 3.2.1. Accuracy and Precision

Across ERNDIM samples, the mean accuracy of C0 was 107.6% over a concentration range of 3.3–71.2 µmol/mmol creatinine. Comparison of response coefficients obtained via external calibration and standard addition indicated negligible matrix effects (<5%). Intra-assay variation for C0 and creatinine was <20%, while inter-assay variation was <18%. Normalization of C0 concentrations to creatinine resulted in intra-assay variability below 5% and inter-assay variability of 18.5%, 5.3%, and 5.7% for QC-L, QC-M, and QC-H, respectively.

#### 3.2.2. Selectivity and Specificity

C0 and creatinine were quantified using analog stable isotope-labeled IS. Butylation of C0 and underivatized creatinine generated two distinct MRM transitions (Table 1), allowing designation of quantifier and qualifier ions. Quantifier ions were selected for higher sensitivity, showing sensitivity enhancement factors of ~20 for C0 and ~3 for creatinine in DUS compared to the qualifier ions. Monitoring both transitions ensured accurate and specific detection of each compound.

#### 3.2.3. Linearity

Excellent linearity was observed for C0 (R^2^ = 0.998 in urine, R^2^ = 0.9994 in DUS; 3784 µmol/L) and creatinine (R^2^ = 0.997 in urine, R^2^ = 0.9995 in DUS; 126 mmol/L).

#### 3.2.4. Sensitivity and Carry-Over

Using quantifier ions, the LLOD and LLOQ in DUS were 0.02 µmol/L and 0.07 µmol/L for C0 and 0.01 mmol/L and 0.04 mmol/L for creatinine, respectively. These values represent sensitivity estimates based on the predefined S/N criteria and were not directly assessed as quantitative levels during method validation. The experimentally assessed calibration ranges were 4–800 µmol/L for C0 and 0.5–30 mmol/L for creatinine. No significant carry-over was detected following injections of high-concentration samples.

#### 3.2.5. Process Efficiency

Process efficiency in DUS urine spots ranged from 98.2–107.9% for C0 (mean 101.4%) and 93.2–112.3% for creatinine (mean 99.2%).

#### 3.2.6. Stability

The maximum CV for processed DUS samples for 28 days was 2.5% for C0 and 2.7% for creatinine under storage at 4 °C. No significant changes were observed when samples were stored at room temperature under ambient light for up to 10 days. To simulate potential shipping conditions, stability was evaluated at 40 °C for 7 days, yielding a maximum CV of 3.9% for C0 in DUS (µmol/mmol creatinine) in QC M. Freeze–thaw experiments over eleven cycles showed a maximum CV of 15.6% for C0 in DUS (µmol/mmol creatinine) in QC L.

#### 3.2.7. Cross-Validation

**Figure 3:**
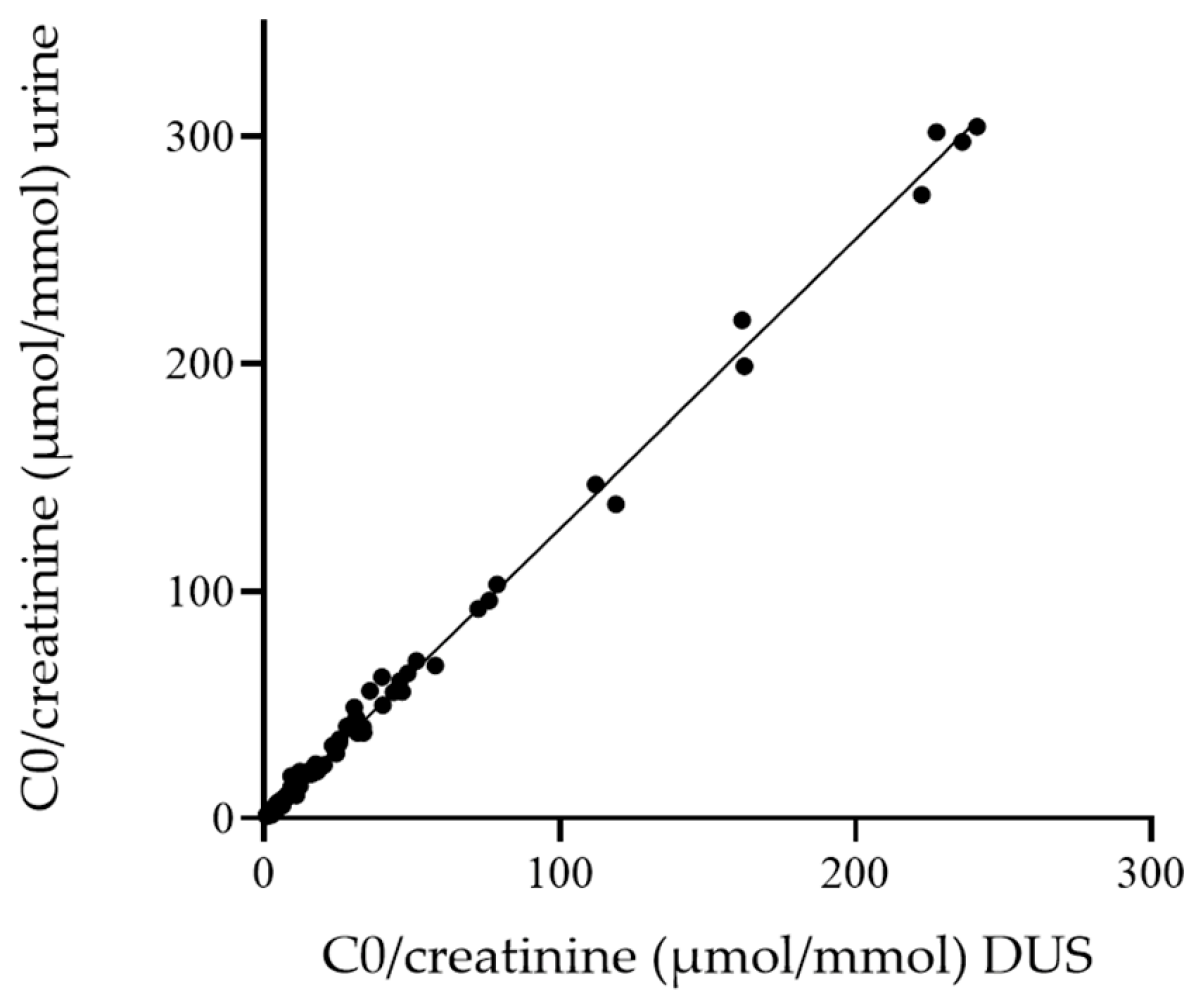
Correlation between C0/creatinine measurements in urine samples and uncorrected DUS measurements (µmol//mmol). The solid line represents the fitted linear regression line: *y* = 1.2719*x* + 0.2394 (R^2^ = 0.9967, *n* = 102).

C0 excretion (µmol/mmol creatinine) measured using the present FIA-MS/MS method in liquid urine showed strong correlation with routinely used techniques (R^2^ = 0.9915, *y* = 1.0149*x* + 2.69; *n* = 41), confirming agreement with the Jaffé method and previously reported ESI-MS/MS measurements [18]. Analysis of 102 paired DUS and liquid urine samples demonstrated a strong correlation between matrices (C0: R^2^ = 0.9962, *y* = 1.0176*x* − 4.8488; creatinine: R^2^ = 0.9727, *y* = 1.1694*x* + 0.0632). When expressed per mmol/L creatinine, C0 values in DUS were lower (Figure 3), necessitating a correction factor of 1.27 to estimate liquid urinary concentrations. This correction factor was calculated as the mean of the individual ratios obtained from 102 paired urine and DUS samples, with each ratio calculated by dividing the analyzed urinary C0 concentration (µmol/mmol creatinine) by the analyzed C0 concentration (µmol/mmol creatinine) in the corresponding DUS. Following this adjustment, Bland–Altman analysis showed no significant difference between DUS and liquid urine measurements (*p* = 0.37). The mean bias was −0.33 µmol/mmol, with a 95% confidence interval of −1.05 to 0.40 and 95% limits of agreement ranging from −7.57 to 6.92. The analysis covered a concentration range of 0.98–304.9 µmol/mmol creatinine, demonstrating good agreement between the two matrices.

## 4. Discussion

This study demonstrates that C0 excretion can be reliably quantified in DUS using a minimal sample volume (6 mm punch ~1.9 µL), with results closely matching those obtained from liquid urine (Figure 3). Normalization to creatinine proved essential to correct for variability in urine dilution, spot deposition, and the amount of urine applied to the filter paper inherent to DUS. The filter paper matrix effectively stabilizes both analytes, enabling accurate quantification after extended storage (at least 10 days at ambient temperature and at least 7 days at 40 °C), supporting the robustness of DUS as a sampling matrix for neonatal screening applications.

Despite overall agreement, C0 excretions (µmol/mmol creatinine) were consistently lower in DUS than in liquid urine, requiring application of a correction factor (1.27). This bias is likely explained by differences in extraction efficiency (Figure 1), with carnitine showing lower recovery than creatinine, and by uncertainty in the volume correction based on spot area, which assumes homogeneous analyte distribution and uniform spot thickness. These findings highlight the importance of matrix-specific calibration when translating DUS measurements to clinically interpretable values.

In NBS, low C0 concentrations in DBS are frequently caused by maternal carnitine deficiency rather than by neonatal metabolic abnormalities [19]. Carnitine concentrations in DBS reflect both the maternal status (by including prenatal carnitine transfer and resulting neonatal carnitine stock) as well as neonatal metabolism, whereas C0 excretion more directly reflects neonatal carnitine metabolism. Thus, assessment of urinary C0 excretion provides critical complementary information by which abnormal carnitine handling in the neonate can potentially be distinguished from low carnitine status in the neonate due to deficiency in the mother. Previous work demonstrated that urinary C0 excretion, either as a urine-to-plasma ratio or as a C0/creatinine excretion value (cut-off: 15.2 µmol/mmol creatinine at ~1 month of age), can discriminate true PCD cases from false-positive referrals [1]. However, this threshold was established in a selected population with persistently low C0 concentrations (i.e., abnormal in the second DBS that was collected several days after carnitine was shown to be low in the first DBS). The applicability of C0 excretion as a standalone diagnostic criterion following an abnormal first screening depends on postnatal C0 dynamics. Analysis of 859 newborns in whom carnitine was low in the first DBS showed that C0 concentrations in a repeat DBS increased substantially within the first days of life (Supplementary Data, Figure S1), consistent with dietary intake and metabolic adaptation as described previously [20]. In some newborns, there was a large increase (up to 7 times) in DBS carnitine concentration in a second compared to the first DBS, even when the second sample was collected shortly after the first.

The analytical performance demonstrated in this study supports the integration of DUS as an additional analytical dimension within existing NBS workflows. Incorporation of DUS alongside the DBS would enable parallel assessment of systemic C0 concentrations and urinary excretion, thereby providing complementary biochemical information. From an analytical perspective, this dual-matrix approach enhances diagnostic resolution by combining two independent yet physiologically related measurements, which may improve classification performance and reduce uncertainty. Similarly, carnitine excretion may facilitate detection of PCD cases in which plasma C0 concentrations fall within reference intervals at the time of referral [21], highlighting its role as an orthogonal analytical marker.

Although no false negatives have been reported in the Netherlands, the cut-off value of C0 is relatively low in relation to other screening programs [22]. However, adjustments of cut-off values, often guided by previously reported false-negative cases [23], may increase screening sensitivity at the expense of specificity. Implementation of a combined DBS–DUS approach offers a means to mitigate this trade-off. By integrating measurements from two distinct matrices, it becomes possible to refine decision thresholds and improve overall screening performance.

From a practical perspective, DUS offers several advantages: non-invasive sampling, compatibility with home collection, and stability during transport without cold-chain requirements. Population-based feasibility has been demonstrated in established neonatal urine screening programs employing similar methodologies [24,25]. In these program designs, filter paper cards are sent to parents to insert between a dry diaper and the baby’s skin at home. Accordingly, the analytical performance of filter paper applied directly to a diaper was evaluated, demonstrating acceptable recovery and reproducibility.

Several limitations must be addressed prior to implementation. This study used liquid urine applied to filter paper rather than prospectively collected DUS, and routine home sampling may introduce variability, including fecal contamination and exposure to exogenous substances (e.g., skincare products). In addition, the lack of visual contrast in DUS necessitates UV-guided punching, which may complicate high-throughput processing. Future work should evaluate integration into existing NBS infrastructures and assess robustness under routine collection conditions.

In conclusion, DUS provides a robust and analytically sound matrix for the quantification of C0 excretion. Its potential integration into NBS follow-up strategies, in combination with a second DBS, may offer a promising approach to support diagnostic evaluation and optimize referral pathways for PCD.

## Figures and Tables

**Table 1:** Optimized MRM parameters for C0 and creatinine.

Metabolite	Used as	Transition	Cone (V)	Collision (eV)
C0	quantifier	218.2 > 103.0	15	16
C0	qualifier	218.2 > 84.9	15	25
D_3_-C0	IS quantifier	221.2 > 103.0	15	16
D_3_-C0	IS qualifier	221.2 > 84.9	15	25
Creatinine	quantifier	113.9 > 44.0	25	14
Creatinine	qualifier	113.9 > 86.0	25	10
D_3_-Creatinine	IS quantifier	116.9 > 47.0	25	14
D_3_-Creatinine	IS qualifier	116.9 > 89.0	25	10

## Data Availability

The raw data supporting the conclusions of this article will be made available by the authors upon request.

## Supplementary Materials

The following supporting information can be downloaded at https://www.mdpi.com/article/10.3390/ijns12040084/s1, Figure S1: C0 carnitine (µmol/L) in the second DBS.

## Abbreviations

The following abbreviations are used in this manuscript:

PCD	Primary carnitine deficiency
OCTN2	Organic Cation Transporter Novel 2
NBS	Newborn screening
C0	Free carnitine
DUS	Dried urine spot
FIA–MS/MS	Flow injection analysis–tandem mass spectrometry
LLOD	Lower limits of detection
LLOQ	Lower limits of quantification
CV	Coefficient of variation
DBS	Dried blood spot
PPV	Positive predictive value
IS	Internal Standard
MeOH	Methanol
ACN	Acetonitrile
QC	Quality control
MRM	Multiple reaction monitoring
ESI	Electrospray ionization
ERNDIM	European Research Network for the Evaluation and Improvement of Screening, Diagnosis, and Treatment of Inherited Disorders of Metabolism
