# Supplementary material for: Dried Urine Spots for Quantification of C0 (Free) Carnitine Excretion: A Validated Method for Follow-Up and Confirmatory Testing of Primary Carnitine Deficiency
Source: Int J Neonatal Screen. 2026 Oct 12;12(4):84. doi: 10.3390/ijns12040084 (PMC13692506; doi:10.3390/ijns12040084)
Supplement: Supplementary file 1 [file IJNS-12-00084-s001.zip › IJNS-4526769-supplementary.pdf]

Supplementary data

Rate of C0 increase in a Second DBS

The Dutch NBS program for PCD follows a two-step protocol: newborns with an initial DBS C0 ≤ 5 µmol/L are offered a second DBS collection and only if the C0 is low in this retest sample, the newborn is referred for clinical evaluation. To investigate the changes in DBS C0 concentrations between the first and second DBS, we retrospectively analyzed all cases between July 2009 and December 2024 in which the initial DBS C0 ≤ 5 µmol/L (cut-off).

Study Population and Data Collection

All neonates with an initial DBS C0 < 5 µmol/L during this period were included. Data were obtained from the registry of the Department for Vaccine Supply and Prevention Programs of the Rijksinstituut voor Volksgezondheid en Milieu (RIVM-DVP). The study was approved by the Data Applications Committee of RIVM-DVP. Each dataset contained paired first and second DBS C0 concentrations. Only data from neonates whose parents had not objected to the use of NBS data for research were included. Datasets were pseudonymized prior to analysis.

Results

A total of 900 newborns had initial C0 ≤ 5 µmol/L, including 163 (18%) at the upper reference limit (5 µmol/L) and two <1 µmol/L. For forty-one infants (4.6%) no second DBS was obtained because of parental refusal, loss to follow-up, hospitalization, known PCD family history, known secondary C0 deficiency, or death. Among the remaining 859 newborns, the median interval between the first and second DBS was 3 days. Collection days for the second DBS were distributed as follows: day 1 (*n*=26), day 2 (*n*=278), day 3 (*n*=152), day 4 (*n*=173), day 5 (*n*=120), day 6 (*n*=51), and ≥ day 7 (*n*=59; latest at 52 days). Later collection times correlated with higher C0 concentrations (Supplemental, Figure 1). Persistently low C0 (≤ 5 µmol/L in both DBS) concentrations were observed in 206 newborns (24%), who were subsequently referred for clinical evaluation. These data provide context for interpreting DUS measurements and highlight the variability in early postnatal C0 dynamics, supporting utility of DUS as a complementary analytical matrix in NBS follow-up.

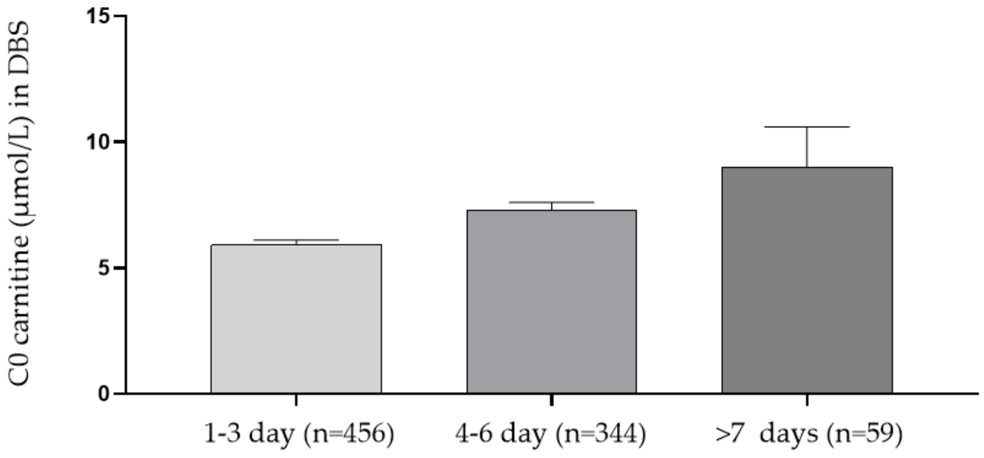

Figure S1. C0 carnitine (µmol/L) in the second DBS collected 1–3 days, 4–6 days, and ≥7 days after the first DBS. Data are presented as median ± 95th percentile, with sample sizes indicated on the x-axis.
